# Stimulus awareness is associated with secondary somatosensory cortex activation in an inattentional numbness paradigm

**DOI:** 10.1038/s41598-023-49857-w

**Published:** 2023-12-19

**Authors:** Antje Peters, Maximilian Bruchmann, Torge Dellert, Robert Moeck, Insa Schlossmacher, Thomas Straube

**Affiliations:** 1 Institute of Medical Psychology and Systems Neuroscience, University Hospital Münster, Von-Esmarch-Straße 52, 48149 Münster, Germany; 2 Otto Creutzfeldt Center for Cognitive and Behavioral Neuroscience, University of Münster, 48149 Münster, Germany

**Keywords:** Consciousness, Perception

## Abstract

While inattentional blindness and deafness studies have revealed neural correlates of consciousness (NCC) without the confound of task relevance in the visual and auditory modality, comparable studies for the somatosensory modality are lacking. Here, we investigated NCC using functional magnetic resonance imaging (fMRI) in an inattentional numbness paradigm. Participants (N = 44) received weak electrical stimulation on the left hand while solving a demanding visual task. Half of the participants were informed that task-irrelevant weak tactile stimuli above the detection threshold would be applied during the experiment, while the other half expected stimuli below the detection threshold. Unexpected awareness assessments after the experiment revealed that altogether 10 participants did not consciously perceive the somatosensory stimuli during the visual task. Awareness was not significantly modulated by prior information. The fMRI data show that awareness of stimuli led to increased activation in the contralateral secondary somatosensory cortex. We found no significant effects of stimulus awareness in the primary somatosensory cortex or frontoparietal areas. Thus, our results support the hypothesis that somatosensory stimulus awareness is mainly based on activation in higher areas of the somatosensory cortex and does not require strong activation in extended anterior or posterior networks, which is usually seen when perceived stimuli are task-relevant.

## Introduction

Neural correlates of consciousness (NCCs) are defined as neural activity sufficient to generate conscious experience^1^. NCCs have been investigated in a number of studies, mainly in the visual modality^1–3^. Consciousness theories disagree about when and where NCCs occur in the brain. Theories such as the recurrent processing theory (RPT)^4,5^ expect NCCs in sensory areas and at relatively early processing stages. Other theories, such as the global neural workspace theory (GNWT)^6,7^, postulate a “broadcasting” mechanism across the brain that causes stimuli to become aware. This mechanism is assumed to be strongly associated with the activity of frontoparietal regions occurring after the initial perceptual processing^6,8,9^. However, GNWT has been challenged by findings suggesting that strong and extended activations of frontoparietal networks during stimulus awareness are associated with the task relevance of stimuli and associated processes, including decision-making and report^2,10–16^. One experimental possibility to control for task-related confounds are studies in which stimuli are task-irrelevant, and awareness is tested by unexpected questions after the experiment.

By using inattentional blindness paradigms, it has been shown that aware perception of visual stimuli is associated with activation mainly in visual areas but not with a strong, late activation outside these visual areas^10,12–14^. Similarly, in the auditory modality, controlling for task effects in inattentional deafness paradigms showed that early but not late ERP effects are related to stimulus awareness^11^. In contrast to those inattentional studies that investigated NCCs to task-irrelevant stimuli in vision and audition, there is no corresponding inattentional numbness study in the somatosensory modality. While two behavioral studies used an inattentional numbness design and showed reduced awareness of somatosensory stimuli depending on concurrent tasks^17,18^, these studies comprised a report about the stimulus awareness after each trial. Thus, whether maintaining inattentional numbness across many trials in suited no-report designs is possible in at least some participants has not been investigated yet. Such designs would comprise parallel neuroscientific studies in other modalities, in which perception of stimuli was (i) task-irrelevant, (ii) assessed after the experiment, and (iii) related to brain responses by comparing aware and unaware participants^10,12–14^.

Nevertheless, there are several neuroscientific studies on conscious stimulus perception in the somatosensory modality (e.g., with (M)EEG^19–25^, intracranial recording^26^ and fMRI^27,28^). The studies mainly support the hypotheses that responses at a latency of around 140 ms are probably associated with activation in the secondary somatosensory cortex (SII) and correlate with somatosensory awareness, while earlier activations of the primary somatosensory cortex (SI) mainly represent stimulus intensity but not stimulus awareness (cf., e.g.^19–22,24,25,29–31^; but see^23^).

Several studies also suggest that activation of frontoparietal areas is critically involved in perceptual awareness of somatosensory stimulation^26–28^. However, in these studies, stimuli were task-relevant, so findings might reflect task relevance and associated post-perceptual processes instead of stimulus awareness. A recent fMRI study that aimed to control for report-related effects by a specific modeling approach found that effects of stimulus awareness are restricted to the secondary somatosensory cortex (SII), while activation in the fronto-parietal cortex reflected target relevance of somatosensory stimuli^29^.

However, the latter study’s result remains to be replicated using other experimental designs, such as inattentional numbness. If a suited number of participants remains unaware of completely task-irrelevant, repeatedly presented somatosensory stimuli, neural correlates of somatosensory consciousness regardless of task-relevance and report can be investigated similar to inattentional blindness or inattentional deafness designs in recent NCC studies^12,13,32,33^. The current fMRI study investigated brain activation during a novel inattentional numbness experiment to address this question. In this paradigm, weak somatosensory stimuli were applied while participants had to solve a demanding visual task. Half of the participants was informed about the task-irrelevant weak somatosensory stimuli, while the other half believed that stimuli below the detection threshold would be applied. After the experiment, all participants were unexpectedly asked whether they had consciously perceived the completely task-irrelevant somatosensory stimuli during the visual task. We expected that the visual task would inhibit awareness of the weak somatosensory stimuli depending on prior information, with more numbness cases in uninformed than in informed participants. More importantly, we hypothesized that awareness effects would be mainly observed in somatosensory areas, above all SII, but not in extended fronto-parietal areas.

## Methods and materials

### Subjects

The sample, which was also part of a previous study on somatosensory processing and therefore described already elsewhere^34^, consisted of 47 participants recruited from the local student community of the University of Münster via public advertisements. All had normal or corrected-to-normal visual acuity and no psychiatric or neurologic illness history. Participants provided written informed consent before the experiment and received monetary compensation (10 €/h). All procedures were approved by the ethics committee of the Medical Faculty of the University of Münster and were conducted following the Declaration of Helsinki (6th revision). Three participants could not be included in the analysis due to technical failures during the experiment. The final sample comprised 44 participants (33 female, 11 male) with a mean age of 24.4 years (SD = 3.6) and a range of 20–35 years.

### Stimuli and experimental paradigm

We used the same or similar procedures of somatosensory stimulation, visual stimulation and experimental parameters as already described in detail in a previous study on visual load and somatosensory processing^34^.

#### Somatosensory stimuli

Somatosensory stimuli were applied via the BIOPAC MP150 system with the stimulation module STM100C using the software AcqKnowledge 5.0 (BIOPAC Systems, Inc.). Two Ag/AgCl skin electrodes were attached to the participants' wrists to stimulate the left nervus radialis superficialis. The left fovea radialis was considered an anatomical landmark to obtain equal stimulation conditions. Via the skin electrodes, a clearly perceptible electric pulse burst (10 ms, consisting of 5 pulses of 2 ms) served as the task-irrelevant tactile stimulus during the visual task. Before the experiment, individual somatosensory thresholds for each participant were identified by gradually increasing and decreasing the voltage output of the BIOPAC stimulation module and asking subjects to report when they started or stopped to perceive the stimulation until the perception threshold voltage V_c_ was identified. The maximum voltage was 40 V, corresponding to a current of approximately 40 to 0.04 mA for a typical skin resistance of 1 to 100 kΩ^35^. Currents used for the remainder of the experiment corresponded to a voltage of 1.25 times the V_c_ to ensure above-threshold stimulation while keeping stimulus intensity as low as possible. The aim of the stimulation was explained to participants by specific cover stories (see below).

#### Visual stimuli

Throughout the experiment, a white cross that measured 0.9 × 0.9 degrees of visual angle was presented in the center of the screen. Simultaneously, 12 small red dots (RGB = [102, 0, 0]) rotated around the fixation cross on three different paths with radii of 0.7°, 1.3°, and 1.9°, respectively, at a constant velocity of 60°/s. The dots on each trajectory had radii of 0.09°, 0.12°, and 0.15°, respectively. The direction of rotation changed after 8 to 16 stimulus presentations. Occasionally, one of the dots changed color for 200 ms to serve as a target for the visual task, and participants were asked to respond to the color change by pressing a key on the right-hand side of the keyboard. Participants were instructed to keep their gaze on the fixation cross. The presentation was implemented using MATLAB and the Psychophysics Toolbox^36,37^. Participants viewed the stimuli by looking at a mirror attached to the head coil. The image was projected onto a semitransparent screen located at the head end of the scanner with a resolution of 1920 × 1080 pixels and a refresh rate of 120 Hz.

#### Experimental procedure

**Figure 1:**
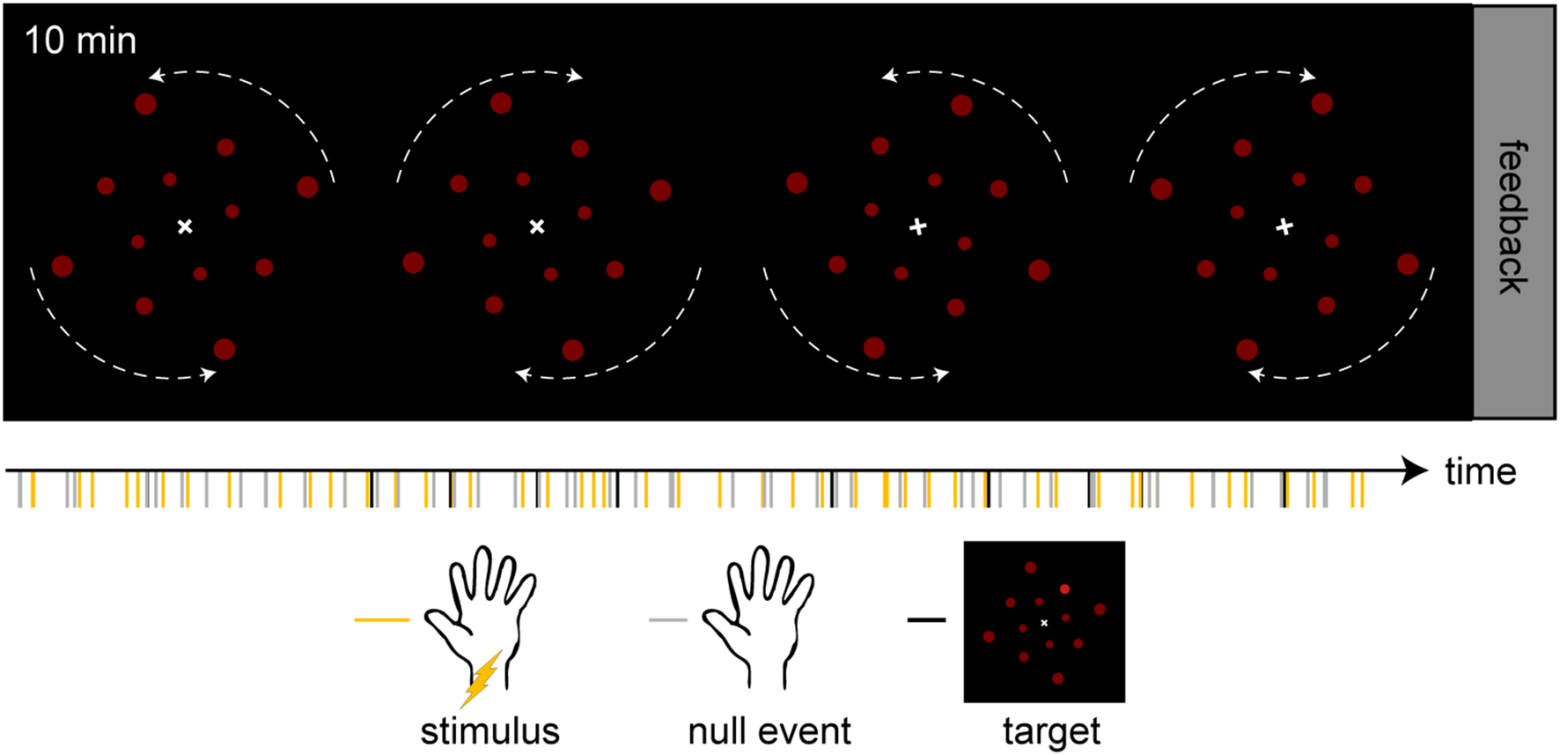
Schematic diagram of the experimental procedure. The illustration shows one of the two runs containing 50 somatosensory stimulus events and 50 null events each. Each run took 10 min. The task comprised the detection of brief color changes as depicted in the bottom row. Rotation changes are indicated with dashed white arrows for illustration purposes. Their actual occurrence was more frequent and variable in timing (see main text for details).

The main experiment consisted of two runs with 50 somatosensory stimuli presentations and 50 baseline events without somatosensory stimulation ("null events"). Stimuli and null events were presented in a pseudo-random sequence, optimized using the Optseq algorithm (http://www.surfer.nmr.mgh.harvard.edu/optseq/), which provides the optimal temporal jitter to increase signal discriminability^38^. Interstimulus intervals varied between 1.8 and 10.8 s (average: 6 s). Visual target stimuli randomly appeared ten times during each run, with an average of one every 60 s. A target stimulus was a 200 ms color change of a randomly selected dot from red to bright red (RGB = [204, 26, 26]). Although no feedback was given during the experimental runs, participants were given information about their accuracy (percentage of correct answers) in the visual task at the end of each 10-min run to motivate their involvement. Participants were also permitted to take breaks between runs as necessary. Figure 1 provides a schematic diagram of the experimental structure.

#### Groups

To test whether stimulus perception expectation affected stimulus reports, participants were split into two groups. Half of the subjects were informed about the presentation of above-threshold somatosensory stimuli at the beginning of the experimental session. However, participants were not informed about the number of applied stimuli and they were told that stimuli would be completely task-irrelevant and should be ignored. The other half of the subjects were presented with a cover story about why electrodes were attached to their wrists: Electrodes were pretended to be attached to perform a skin conductance measurement during the experiment. During this measurement, it was announced that weak currents were applied through the electrodes. To ensure that participants were not irritated by the electrical currents, they had to be applied below the threshold. According to the cover story, the pre-experimental threshold procedure aimed to find the below-threshold stimulation for the experiment. None of the participants expected any further questions regarding the somatosensory stimulation. After the fMRI session, all participants were asked four unannounced questions, which should ensure sufficient sensitivity to identify all participants who detected at least some stimuli. Subjects were first asked whether they perceived anything and then if they perceived stimuli on the left wrist. Afterwards, they were informed that they had been stimulated on the left wrist and asked whether they could estimate the number of stimuli. To prevent an overestimation of unaware participants, e.g., from relying on the last experimental block for their estimate, we additionally asked how many stimuli were perceived per quarter of the experiment. Only if participants reported zero stimuli in all four questions, they were classified as unaware, in other cases as aware. On average, the remaining subjects reported perceiving M = 45.4 stimuli (SD = 83.2) when asked about the total experiment and in sum M = 41.6 stimuli (SD = 47.8) when they estimated stimuli perception per quarter of the experiment.

The mean age of the informed and uninformed groups did not differ significantly (mean_inf_ = 24.3; SD_inf_ = 3.8 years and mean_uninf_ = 24.5; SD_uninf_ = 3.4 years, *p* = 0.804). The percentage of females in each group did not differ significantly (p(females)_inf_ = 81.8%, p(females)_uninf_ = 68.2%, *χ*^2^ = 1.091, *p* = 0.296).

### Behavioural data analysis

Task performance was quantified by each subject's response times (RTs) and hit rate. In cases of a 0% hit rate (3 participants), no response time could be recorded. A 2-way Schreier–Ray–Hare test was performed to compare the performance parameters of all subjects between the aware and unaware groups and between the informed and uninformed groups. We used a χ^2^-test to compare the distribution of aware and unaware participants between the informed and uninformed group.

### Image acquisition and analysis

The image acquisition and analysis procedure is comparable to our previous study on somatosensory processing and procedures have been already described there in detail^34^.

#### FMRI data acquisition

A 3-Tesla Siemens Magnetom Prisma and a 20-channel Siemens Head Matrix Coil (Siemens Medical Systems) were used for fMRI data collection. A high-resolution T1-weighted scan was obtained with 192 slices, a TR of 2130 ms, a TE of 2.28 ms, an FA of 8°, and a voxel size of 1 × 1 × 1 mm within a FOV of 256 × 256 mm. Two datasets were gathered for each participant during the tasks using a T2*-weighted echoplanar sequence sensitive to blood oxygenation level-dependent (BOLD) contrast. The imaging parameters included a TR of 2300 ms, a TE of 30 ms, a FA of 90°, a FOV of 216 × 216 mm, and a voxel size of 3 × 3 × 3 mm. The datasets consisted of 274 volumes with 42 interleaved axial slices (3 mm thickness, 0.3 mm gap). The slices were angled at approximately 25° from the anterior–posterior commissure plane to reduce susceptibility artifacts in anterior inferior brain areas. Before functional imaging, a shimming field was utilized to decrease magnetic field inhomogeneity.

#### fMRI data preprocessing

We used MATLAB 9.7 (MathWorks) with SPM12 version 7771 (The Wellcome Centre for Human Neuroimaging, UCL Queen Square Institute of Neurology, London, UK; https://www.fil.ion.ucl.ac.uk/spm/software/spm12/) and the Data Processing and Analysis of Brain Imaging (DPABI) 6.0 toolbox^39^ for preprocessing. The first five data volumes were excluded for spin saturation effects. The remaining volumes were subjected to slice time correction and realigned using a linear transformation with six parameters (rigid body). The anatomic and functional images were then coregistered. We used DARTEL^40^ for nonlinear spatial normalization of the data to Montreal Neurological Institute (MNI) standard space. Finally, data were spatially smoothed with a 6 mm full-width at half-maximum Gaussian kernel.

#### fMRI data analysis

We removed slow signal drifts from the data using a high-pass filter with a cutoff of 128 s. To account for autocorrelations, we utilized the SPM pre-whitening method FAST^41^ recommended by Olszowy and colleagues^42^. We performed a first-level data analysis using a general linear model (GLM) for each participant. The GLM design matrix included two predictors of interest: somatosensory stimuli and null events. The visual target presentation, target responses, and six head movement parameters were defined as predictors of no interest. Onsets of experimental predictors were convolved with a 2-gamma hemodynamic response function to model the BOLD signal change for each predictor. Based on the first-level analysis, we computed contrast images of the β-estimates for the stimulus main effect (stimulus—null event) for each participant. In a second-level analysis, we identified clusters with different activation in the stimulus condition compared to the null event across participants. Moreover, we identified clusters with different activation in the aware compared to the unaware group for this contrast. Analyses were performed across the whole brain based on cluster-based permutation tests with a voxel-level-threshold of α_voxel_ = 0.001, 5000 permutations, and cluster-mass statistics using PALM^43^. The p-values were FWER-corrected^44^. Only clusters that passed the cluster threshold α_cluster_ < 0.05 were considered significant.

## Results

### Behavioral data

#### Awareness ratings

17 of 22 uninformed subjects and 17 of 22 informed subjects were aware of the stimuli (χ^2^ = 0, *p* = 1, i.e., identical distribution of awareness). Thus, prior information did not affect numbness outcomes. For further analysis and due to the small number of unaware subjects, we used a combined aware group (34 subjects) and an unaware group (10 subjects).

#### Reaction times and hit rates

For reaction times (mean = 678; SD = 375 ms), the result of the nonparametric Schreier–Ray–Hare test indicated no significant main effect of awareness (*H(1)* = 0.618, *p* = 0.432), no significant main effect of information (*H(1)* = 0.122, p = 0.726), and no significant interaction between awareness and information (*H(1)* = 0.083, *p* = 0.774). For hit rates (mean = 60.5; SD = 31.8), the result of the nonparametric test indicated no significant main effect of awareness (*H(1)* = 0.383, *p* = 0.536), no significant main effect of information (*H(1)* = 0.294, *p* = 0.588), and no significant interaction between awareness and information (*H(1)* = 0.302, *p* = 0.583).

### Functional MRI data

#### Main effect of stimulation

**Figure 2:**
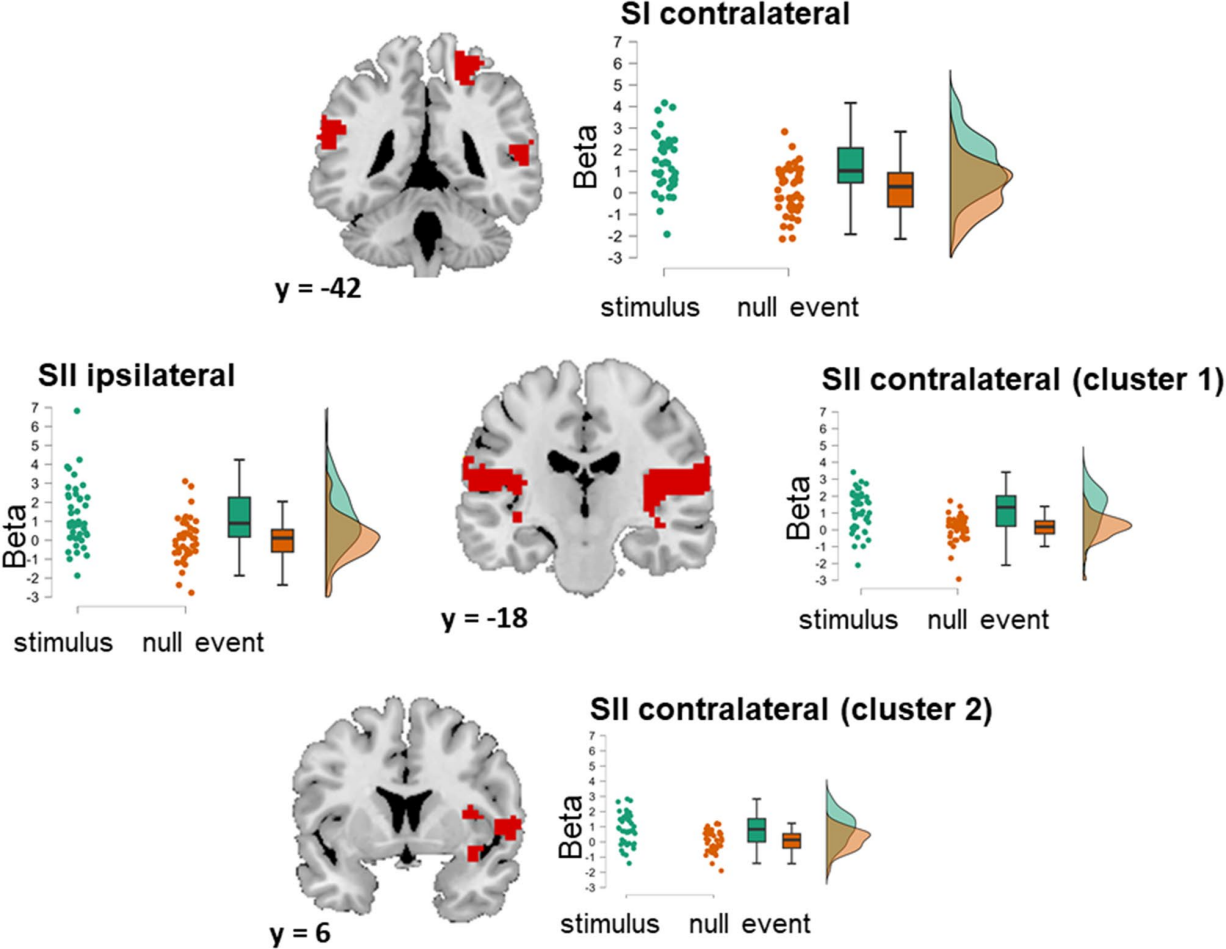
Clusters of increased activity in the stimulus–null event contrast for all participants. Displays of the clusters are accompanied by raincloud plots of the individual data points in each condition, comprising a jittered scatter plot of the cluster average of the betas per subject, a box-and-whisker plot, and a density plot of the same data. Note that the y-axis of the plot goes below zero.

We found four clusters of significantly increased activation in the stimulus compared to the null event condition across all participants: one cluster in the contralateral SI (peak *t* = 6.47; x, y, z = 24, − 42, 66; cluster size: 170 voxels), one cluster in the ipsilateral SII (peak *t* = 5.66; x, y, z = − 39, − 9, − 6; cluster size: 392 voxels) and two in the contralateral SII (cluster 1: peak *t* = 8.72; x, y, z = 39, − 18, 18; cluster size: 690 voxels, cluster 2: peak *t* = 5.19; x, y, z = 60, 6, 3; cluster size: 66 voxels). A visualization of the clusters and mean beta values for the stimulus vs. null event condition are presented in Fig. 2.

#### Awareness effect

**Figure 3:**
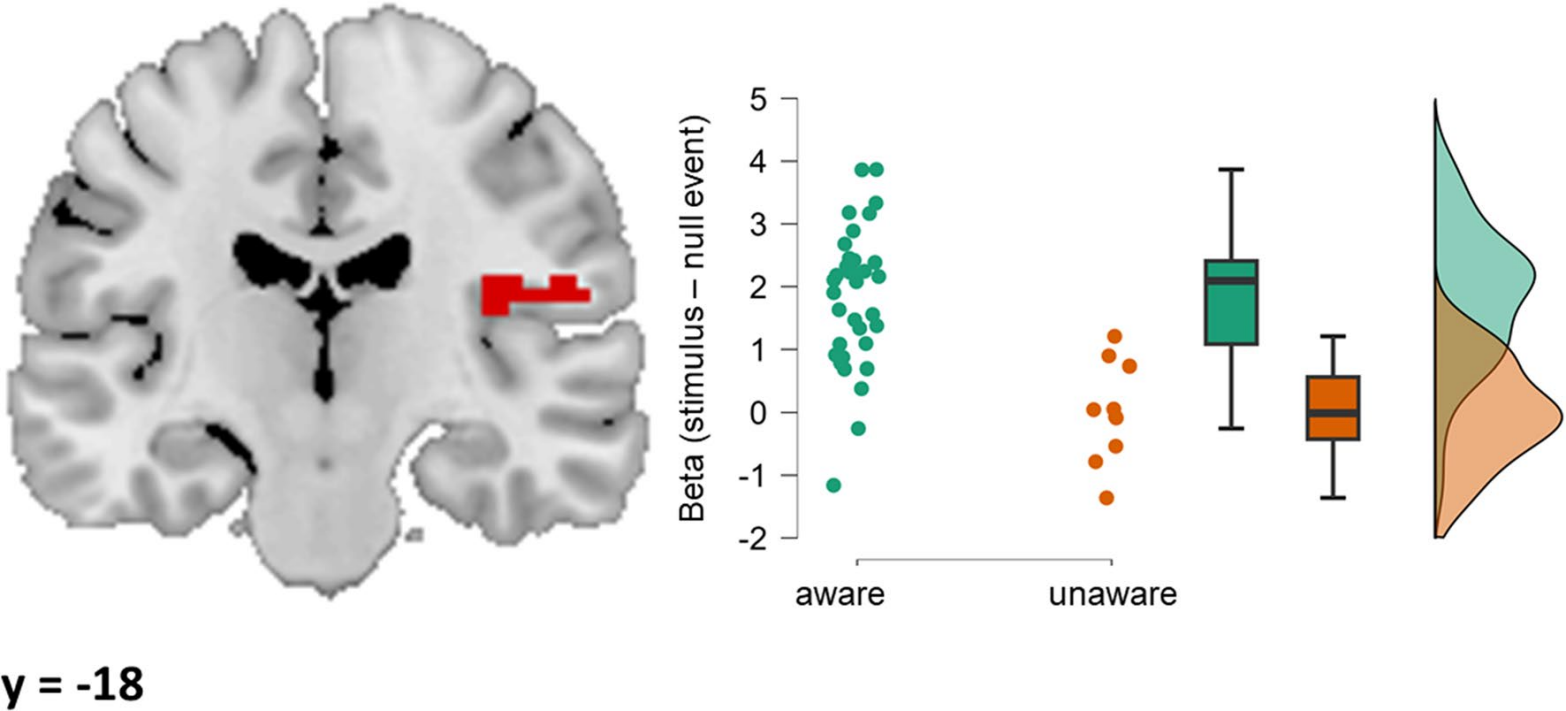
Cluster of increased activity in the aware compared to the unaware group. The display of the cluster is accompanied by a raincloud plot of the individual data points in the aware and the unaware group, comprising a jittered scatter plot of the cluster average of the betas per subject, a box-and-whisker plot, and a density plot of the same data. Note that the y-axis of the plot goes below zero.

We found significantly increased activation in aware compared to unaware participants in the right SII, i.e., contralateral to the stimulation (peak *t*-value = 4.65; x, y, z = 36, − 18, 18; cluster size: 66 voxels). The clusters and associated beta values for the aware and unaware group are visualized in Fig. 3.

**Figure 4:**
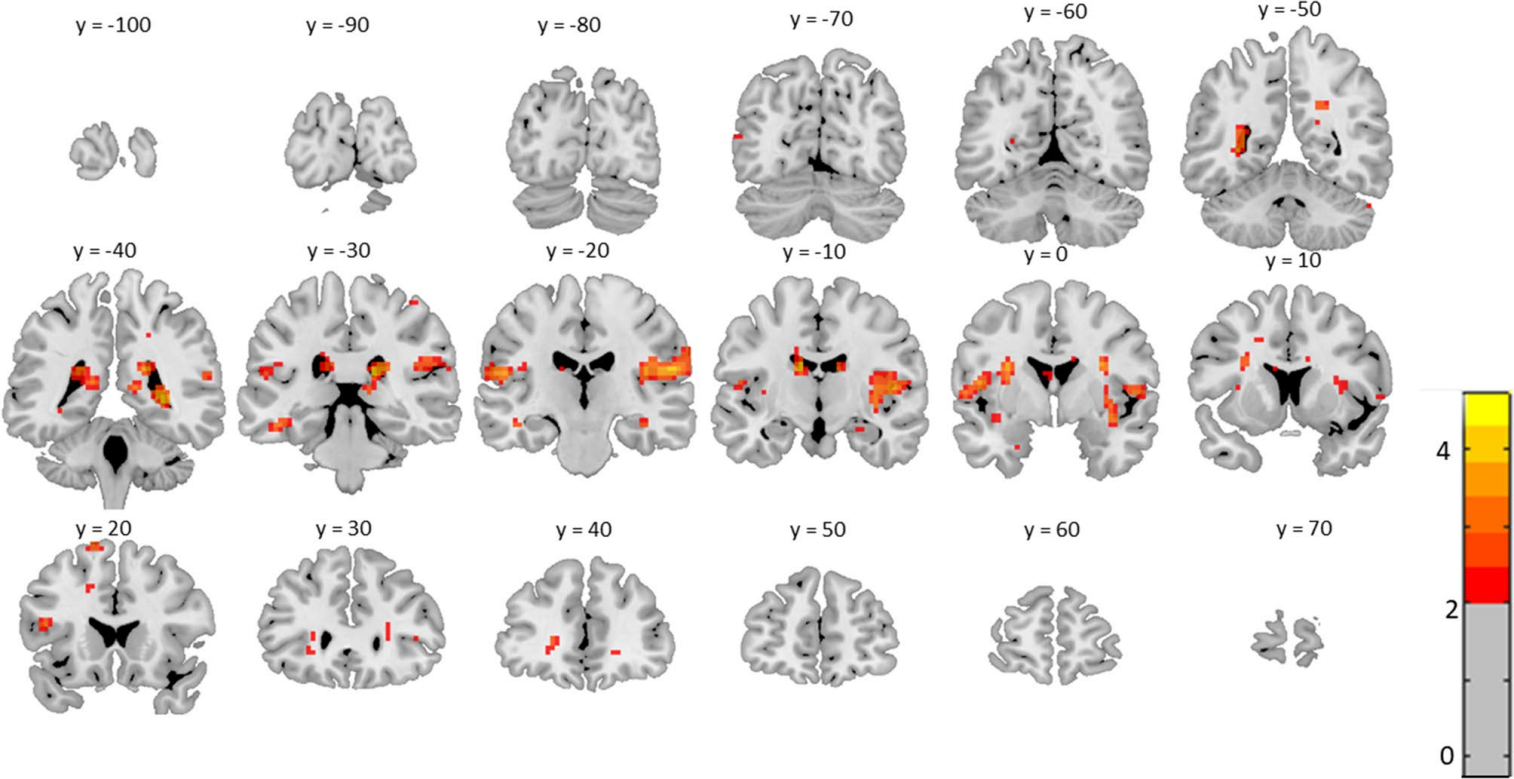
Displays of t-maps thresholded at a voxelwise *p* < 0.05 (*t* > 2.00) for the aware vs. unaware contrast. While increased t-values are seen, the permutation analysis only revealed significant effects in the right (i.e., contralateral) SII.

No other brain area showed a significant difference between aware and unaware participants. To estimate whether this might represent a threshold effect, we additionally investigated brain activation at a more lenient voxel-level threshold of α_voxel_ = 0.05. Even with this threshold, the permutation-based analysis did not reveal any effect. Furthermore, for exploratory reasons, Fig. 4 shows voxelwise, uncorrected t-maps thresholded at a voxelwise *p* < 0.05 for the contrast between aware and unaware participants across the whole brain. This map shows clustered effects in the bilateral SII/posterior insula area but no comparable clusters in other areas. Notably, this exploratory figure shows small clusters in the inferior and superior frontal gyrus and anterior insular cortex.

## Discussion

The primary purpose of this study was to investigate brain activation associated with perceptual awareness of somatosensory stimuli in a novel inattentional numbness paradigm. We found significant effects of stimulus awareness in the secondary somatosensory cortex on the hemisphere contralateral to the stimulus application. On the other hand, we found no significant effects in other areas, including the primary somatosensory cortex or frontoparietal areas.

Our study aimed at isolating awareness-related brain activity from task-related post-perceptual activity due to the task-irrelevance of somatosensory stimuli and surprising awareness ratings only after the experiment. In the visual and auditory modality, several studies using inattentional blindness or deafness or other suited task designs that disentangle awareness and post-perceptual processes suggest that the main NCC is found in the activation in sensory areas^10–14^. In contrast, a robust and late activation outside these areas strongly depends on task requirements. We want to note that we do not exclude the possibility of weaker effects outside SII, which might correspond to our exploratory findings, as discussed below. However, our study supports the central role of SII in understanding somatosensory awareness.

Previous studies that investigated NCC for somatosensory stimuli also showed that activation in the secondary somatosensory cortex (SII), but not earlier activation of the primary somatosensory cortex (SI), correlates with somatosensory awareness (cf.^19–22,24,25,29–31^; but see^23^). However, several studies also suggest a role of activation in frontoparietal areas in perceptual awareness of somatosensory stimulation^26–28^. It should be noted that none of these latter studies controlled for the brain responses associated with the report of perceptual awareness. However, a recent study^29^ used a modelling approach in combination with a dual-task design to disentangle report-related effects from somatosensory awareness by presenting somatosensory stimuli at different intensities and under different tasks. This study found that stimulus awareness was associated with activation in the secondary somatosensory cortex. In contrast, activation in SI was mainly associated with stimulus intensity and activation in frontoparietal areas with report-related confounds of detection designs. Our findings replicate the main conclusions of this previous study using another experimental approach that allowed us to control for task-relevance effects when investigating brain activation associated with somatosensory stimulation awareness.

Remarkably, our additional exploratory analyses with lenient thresholds did not reveal prominent clusters outside SII, including typical frontoparietal areas. Whether the small clusters seen in our exploratory Fig. 4 are reliable has to be answered by future research. Possible awareness effects in several frontoparietal areas may be associated with high interindividual heterogeneity^45^, requiring other analytical approaches. Nevertheless, even if our exploratory data might provide tentative evidence for the role of specific frontal areas in conscious perception, there is no evidence for a robust and strong frontoparietal awareness effect, typically seen in designs in which report and awareness effects are not separated^26–28^.

Our as well as other recent findings^29^ show that awareness effects are only reliably observed in the contralateral SII, which supports a role of a higher somatosensory cortex area beyond SI in understanding the NCC of somatosensory stimulation. While SI has a somatotopic structure^46^ and processes information from the contralateral half of the body^47,48^ on a basic level, higher somatosensory stimulus processing occurs in SII and is less lateralized than SI^49^. SII is involved in further spatial processing of stimuli and cognitive processes, such as integrating information from different modalities and body parts^50–53^. Our findings are also in accordance with EEG data, showing that the initial response in SI around 50 ms is not related to stimulus awareness. In comparison, a later response around 140 ms, associated with activation of SII, correlates with stimulus awareness^19–22,24,25^. While effects seem to be mainly found in contralateral SII, we would like to note that our exploratory Fig. 4 also suggests awareness effects in the ipsilateral SII, which might not become significant due to the applied thresholds.

Our findings are consistent with theories suggesting a priority role of sensory areas in conscious perception^1,54–57^, but inconsistent with theories that include the need for strong, widespread frontoparietal activation as a core element^6,8,9^. As discussed, we cannot exclude the possibility that weaker and more variable effects in frontoparietal areas correlate with stimulus awareness. However, such weak effects also require reformulating strong ignition accounts, as predicted by the GNWT^8^. Furthermore, the causal role of such effects in stimulus perception has to be shown in future studies. These effects might represent several further aspects of stimulus processing, including orienting responses or activation of stimuli-related associative networks. However, regardless of the specific brain area and/or spatio-temporal brain activation pattern, it remains a challenging task for future studies to probe the causal, modulating, or simply correlating nature of specific brain responses associated with awareness of stimuli^15^.

Somewhat surprisingly, we found no effect of previous information on the detection of stimuli. We hypothesize that it is difficult not to perceive the stimuli even in the uninformed group, as it has been shown that tactile stimuli have an exceptionally high potential to attract attention^58–60^. This finding underlines the difficulty of hiding a clearly perceptible tactile stimulus across many trials. Furthermore, even the uninformed group was aware of the stimulation and expected below-threshold stimulation. It remains unclear whether a design with a changed cover story leading to the lack of any expectation or knowledge regarding somatosensory stimuli would change the outcomes.

Nevertheless, in some subjects, the task focus on the visual task induced inattentional numbness regarding the task-irrelevant weak tactile stimuli. Given our study's parameters, this task focus seems more critical than prior information. Differences in attention paid to the visual task in combination with other internal or external attention-distracting factors are responsible for differences in the inattentional numbness outcome, similar to inattentional blindness designs^32^. Behavioural studies showed that a concurrent task reduces awareness of somatosensory stimuli depending on the load of the task^17,18^. However, in contrast to Murphy and Dalton's studies, which are no neuroimaging studies but purely behavioral studies, we could not vary the load and include trial-by-trial ratings, since we needed the same task conditions for aware and unaware subjects to isolate neural correlates of stimulus awareness regardless of load and task-relevance confounds. Thus, our design required (1) many trials (in contrast to single trial studies, which are typically used to separate between aware und unaware subjects), (2) the same experimental conditions (in contrast to load manipulations) and (3) surprising post-experimental questions (in contrast to trial-by-trial ratings, which would allow within subjects comparisons). We did our best to create a design with a high load task that leads to a sufficient number of unaware subjects. We would like to note that many inattentional blindness studies do not vary the load of the task, but nevertheless find that many subjects fail to notice the critical stimulus^11–14^.

Regarding differences between aware and unaware subjects, we cannot completely rule out a role of response criteria or illusory perceptions^53^. However, we carefully asked post-experimental questions to ensure that subjects reported any somatosensory perception. Furthermore, the specific activation in the secondary somatosensory cortex, which agrees with a previous study^29^, speaks against a response criterion difference. We suggest that a combination of other factors, such as attention allocation to the visual task (not necessarily correlated with behavioural data, since different subjects have different capacities) and some individual traits^61^ might explain the results. There is some evidence that inattentional phenomena might be based more on stochastic processes without regard to individual cognitive abilities^62^. However, inattentional phenomena have been found to be related to individual personality traits, for example, openness to experience^61^.

We regard our study as a first study in a challenging research area. Future studies with other and even more difficult main tasks might change the general distribution of aware and unaware participants, and effects might vary depending on prior information. Furthermore, we used an established visual task^10,12–14^ to avoid the simultaneous presence of target and distractor stimuli and to separate the task-relevant and task-irrelevant modalities. Future studies should investigate whether other temporal task parameters and sensory modalities, including somatosensory tasks, increase the number of unaware subjects.

Given the asymmetric distribution of female and male participants in our study, possible effects of the gender asymmetry cannot be excluded. Men are generally more likely to perceive unexpected visual stimuli^63^. In the somatosensory modality, no differences between genders have been found^64,65^. Gender differences in somatosensory processing mainly occur when painful stimuli are applied^65^. Future studies should systematically investigate gender differences regarding somatosensory awareness.

Due the abovementioned difficulty to render repeatedly presented somatosensory stimuli unaware, a main limitation of the study is the modest number of unaware subjects. However, as described above, our findings replicate a previous study that used another experimental approach^29^, and we report that highly significant effects in SII contrast with the absence of significant effects, even at lenient thresholds, in other areas. Nevertheless, future studies should increase the sample size or try to vary experimental conditions to change the balance between aware and unaware subjects. Furthermore, inattentional unawareness designs might be confounded by the effects of inattentional amnesia due to the delayed reporting of stimulus awareness. However, the high memorability of somatosensory stimulation and the clear separation between visual tasks and somatosensory distractors makes the amnesia explanation unlikely in the present study.

## Conclusions

The current fMRI study investigated brain responses associated with awareness of somatosensory stimulation in an inattentional numbness paradigm. Awareness of the stimuli led to increased activation in the secondary somatosensory cortex, while we found no significant effects in other areas, including SI or frontoparietal regions. We conclude that SII activation is the main correlate of somatosensory stimulus awareness.

## Data Availability

The datasets analyzed during the current study are available from the corresponding author upon reasonable request.
